# Microbial-Mediated Differential Regulation of Yttrium Behavior in the Rhizosphere: Blocking Uptake in *Lactuca sativa* L. While Enhancing Bioavailability in *Solanum nigrum* L.

**DOI:** 10.3390/microorganisms14050962

**Published:** 2026-04-24

**Authors:** Yuanjin Cheng, Jingjing Chen, Leqing Liu, Chenhui Tian, Minfei Jian, Weiying Wang

**Affiliations:** Jiangxi Provincial Key Laboratory of Biodiversity Conservation and Resource Utilization, College of Life Science, Jiangxi Normal University, Nanchang 330022, China; impossible0126@outlook.com (Y.C.); 13593629538@163.com (J.C.); 15178148839@163.com (L.L.); 13361619873@163.com (C.T.); jianminfei@jxnu.edu.cn (M.J.)

**Keywords:** plant growth-promoting microorganisms, microbial phytoremediation, remediation of yttrium contamination, rhizosphere microecology

## Abstract

To address yttrium (Y) contamination from ion adsorption mining, this study developed a combined microbial phytoremediation strategy for dual objectives: ensuring crop safety in *Lactuca sativa* and enhancing Y recovery by *Solanum nigrum*. Two specific microbial consortia were constructed from rare earth tailings isolates: inoculant I (bacterial: *Enterobacter* sp., *Serratia* sp., *Bacillus* sp.) applied to *L. sativa*, and inoculant II (fungal: *Penicillium* sp., *Aspergillus* sp., *Talaromyces* sp.) applied to *S. nigrum*. Inoculant I increased *L. sativa* biomass by 26% while reducing Y content in roots and rhizosphere soil solution by 47% and 56%, respectively, potentially through down-regulation of amino acid metabolites. Inoculant II increased Y content in the *S. nigrum* rhizosphere soil solution by 89%, linked to up-regulation of organic acids and coumarin derivatives. Both consortia reduced plant stress markers and enhanced soil enzyme activities. These findings demonstrate that specialized microbial consortia can differentially regulate Y behavior in the rhizosphere—immobilizing it in a crop for food safety, while enhancing its bioavailability for a hyperaccumulator—offering a targeted strategy for managing rare earth element-contaminated agricultural soils.

## 1. Introduction

Rare earth elements (REEs), which encompass the lanthanide series along with scandium and yttrium, are essential components in numerous modern technologies, including renewable energy systems, electronic devices, and defense applications [1]. China is the world’s leading producer and exporter of REEs, with ion adsorption rare earth deposits—predominantly located in seven southern provinces—being particularly important due to their enrichment in medium and heavy REEs such as yttrium. These deposits account for over 80% of global reserves [2]. However, extensive leaching mining over several decades has led to substantial releases of ammonium salts and REEs into surrounding ecosystems [3]. This contamination has been shown to significantly alter soil microbial community structures [4] and may pose health risks through food chain transfer, with potential hepatotoxic and neurotoxic effects documented [5]. Elevated REE concentrations in the environment thus represent both an ecological concern and a loss of valuable strategic resources [6]. Consequently, there is a need for effective and environmentally sustainable remediation approaches for REE-contaminated areas.

Phytoremediation has received increasing attention as a cost-effective and ecologically benign remediation strategy [7]. Two principal approaches have been developed: phytoextraction, which utilizes hyperaccumulator plants to recover REEs, and phytostabilization, which aims to limit crop uptake of these elements [8,9]. Plant growth-promoting rhizobacteria (PGPR) play a critical role in these processes by modifying rhizosphere conditions through the secretion of organic acids, phytohormones, and other bioactive metabolites [10,11]. Depending on the specific microbial strains and plant species involved, PGPR can either enhance plant REE accumulation [12] or suppress its uptake through distinct regulatory mechanisms [13]. Despite increasing recognition of their potential, the mechanisms by which PGPR mediate plant-REE interactions remain incompletely understood.

In this study, microbial strains previously isolated from ion adsorption rare earth tailings were selected. Based on comprehensive characterization of plant growth-promoting traits—including indole-3-acetic acid (IAA) production, siderophore biosynthesis, 1-aminocyclopropane-1-carboxylate (ACC) deaminase activity, and phosphate solubilization—two microbial consortia with distinct functional properties were constructed. Bacterial consortium I, consisting of three bacterial strains exhibiting favorable plant growth-promoting traits, was inoculated into *Lactuca sativa* L. in pot experiments to reduce REE accumulation in vegetable and ensure food safety. Fungal consortium II, comprising three fungal strains with strong plant growth-promoting characteristics and acid-producing capabilities, was inoculated into the hyperaccumulator *Solanum nigrum* L. to enhance REE phytoaccumulation and facilitate subsequent resource recovery. By analyzing yttrium accumulation patterns, plant and rhizosphere physicochemical properties, and changes in microbial community structure, this study sought to elucidate the differential effects and underlying mechanisms of these PGPR consortia on plant yttrium uptake. This work provides a foundation for the application of microbe–plant systems in REE resource recovery and environmental remediation.

## 2. Materials and Methods

### 2.1. Screening of Plant Growth-Promoting Rhizobacteria (PGPR)

A total of 128 microbial strains were isolated from rare earth tailings and evaluated for plant growth-promoting traits using multiple assays. Phosphate solubilization capacity was assessed by observing halo zones on Mengjina inorganic and organic phosphorus media. Siderophore production was determined using Chrome Azurol S (CAS) agar plates. Indole-3-acetic acid (IAA) production was assessed via the Salkowski colorimetric method [14]. ACC deaminase activity was evaluated by culturing strains on DF/ADF media and monitoring bacterial growth curves [15]. Based on comprehensive evaluation of these plant growth-promoting traits, six strains exhibiting superior characteristics were selected as candidate PGPR. Molecular identification at the genus level was performed through 16S rRNA gene (for bacteria) and ITS region (for fungi) amplification and sequencing, followed by BLASTn (https://blast.ncbi.nlm.nih.gov/, accessed on 21 April 2026) homology analysis.

### 2.2. Preparation of Soil Substrate, Organic Fertilizer, Seedlings, and Microbial Consortia

Soil substrate: A mixture comprising 1.5 kg of uncontaminated forest soil, 0.5 kg of river sand, 5 mL of 100 mg·mL^−1^ yttrium solution (resulting in a final soil yttrium concentration of 250 mg·kg^−1^), and 40 g of organic fertilizer was thoroughly homogenized and sterilized by autoclaving at 121 °C for 1 h.

Seedlings: Plant seeds were surface-sterilized and germinated in quartz sand, alternately irrigated with Hoagland nutrient solution and sterile water.

Microbial consortia: Two distinct microbial consortia were prepared from the selected strains. For bacterial consortium I, bacterial strains were cultured overnight in Luria–Bertani (LB) medium with shaking, harvested by centrifugation, washed, and resuspended in sterile water to adjust the optical density at 600 nm (OD_600_) to 1.0 (approximately 10^8^ CFU·mL^−1^). Equal volumes (20 mL) of each bacterial suspension were mixed to obtain consortium I. For fungal consortium II, spores of strains 27, 32, and 33 were collected by adding sterile water to solid potato dextrose agar (PDA) plates and scraping with a spreader. The spore suspension was adjusted to a concentration of 10^8^ spores·mL^−1^ using a hemocytometer, and equal volumes (20 mL) of each spore suspension were mixed to obtain consortium II [16].

### 2.3. Pot Experiment and Soil Solution Collection

Four treatment groups were established: lettuce control (LCK), lettuce with consortium I (LM), *Solanum nigrum* control (SCK), and *S. nigrum* with consortium II (SM). Each pot was filled with one unit of the prepared soil substrate. Two uniform seedlings were transplanted into each pot, with five replicate pots per treatment. Plants in the treatment groups received 60 mL of the respective microbial consortium inoculated into the rhizosphere weekly, while control plants received an equal volume of sterile water. Plants were cultivated under controlled conditions with a 16/8 h light/dark photoperiod and day/night temperatures of 28/22 °C. After 45 d, plants were harvested for subsequent analyses, including collection of rhizosphere soil, roots, and leaves.

Prior to harvest, soil moisture content was equalized across all pots. Rhizosphere soil solution was collected using soil solution samplers (Rhizosphere, Rhizon-MOM, Wageningen, The Netherlands). Sampler tips were moistened for 5 s, inserted into the soil at an angle, and connected to a syringe. A vacuum was created by pulling the syringe plunger and maintained with a wooden block to draw pore water. After approximately 30 min, the accumulated solution was collected. A 10 mL aliquot was acidified with 0.1 mL nitric acid, filtered through a 0.45 μm membrane, and stored at 4 °C for subsequent analysis.

### 2.4. Analysis of Plant Biomass, Yttrium Accumulation, and Physicochemical Properties

Plant fresh and dry weights were recorded. For oxidative stress assessment, fresh leaf samples were collected at harvest, immediately frozen in liquid nitrogen, and stored at −80 °C until analysis. Frozen leaf tissues (0.2 g) were ground to a fine powder in liquid nitrogen using a pre-chilled mortar and pestle, then homogenized in 2 mL of pre-cooled phosphate buffer (0.1 M, pH 7.4). The homogenate was centrifuged at 4000× *g* for 15 min at 4 °C, and the supernatant was used for the determination of proline (Pro) and malondialdehyde (MDA) contents, as well as catalase (CAT), superoxide dismutase (SOD), and peroxidase (POD) activities according to established methods [17], using commercial assay kits (Solarbio, Beijing, China: BC0290 for Pro, BC0020 for MDA, BC0200 for CAT, and BC0170 for SOD) according to the manufacturer’s protocols.

For rhizosphere soil enzyme assays, fresh soil samples were air-dried naturally, passed through a 2-mm sieve, and stored at 4 °C for no more than one week prior to analysis. Soil catalase (S-CAT), soil urease (S-UE), and soil acid phosphatase (S-ACP) were measured using corresponding Solarbio kits (BC0100, BC0125, and BC0145, respectively) following the manufacturer’s instructions. Enzyme activities were expressed as units per gram of soil (U·g^−1^) [18].

For yttrium concentration analysis in plant tissues, accurately weighed 0.2 g of dried, ground, and well-mixed samples were digested with HNO_3_-H_2_O_2_ 8:1, *v*/*v* in a microwave digestion system following a programmed procedure: 150 °C for 2 min, 170 °C for 2 min, and 190 °C for 30 min [19]. Yttrium concentrations in the digested solutions were analyzed by inductively coupled plasma mass spectrometry (ICP-MS). Differences in biomass, yttrium content, enzyme activities, and physicochemical properties were analyzed using SPSS (version 20.0) with least significant difference (LSD) tests at significance levels of *p* < 0.05 and *p* < 0.01.

### 2.5. Microbial Community Analysis

Soil microbial DNA was extracted using the cetyltrimethylammonium bromide (CTAB)/sodium dodecyl sulfate (SDS) method. Following quality assessment by agarose gel electrophoresis, DNA samples were diluted to 1 ng·μL^−1^. Target regions were amplified by PCR using high-fidelity DNA polymerase, and high-throughput sequencing was performed on the NovaSeq 6000 platform (Novogene, Beijing, China). After quality filtering, sequencing data were clustered into operational taxonomic units (OTUs) at 97% sequence similarity using UPARSE (v7.0.1090). Representative sequences were taxonomically annotated against the SILVA database.

All subsequent statistical analyses were performed in R (version 4.3.3). Microbial community diversity and structure were evaluated using alpha diversity indices (Chao1 and Shannon) and principal coordinate analysis (PCoA) based on Bray–Curtis distances. To identify key microbial taxa, two complementary approaches were employed. First, Student’s *t*-test was used to compare the relative abundances of each genus between inoculated and control groups, with taxa exhibiting *p* < 0.05 considered significantly different. Second, based on genus-level abundance tables aggregated from non-rarefied OTU tables, random forest classification models were constructed using the classif.ranger() function from the mlr3 package (version 0.22.0). Nested stratified cross-validation was applied to identify microbial biomarkers contributing significantly to discrimination between groups. Finally, to examine associations between microbes and environmental factors, Spearman’s rank correlation analysis was conducted between the relative abundances of identified differential taxa and simultaneously measured soil physicochemical properties.

### 2.6. Non-Targeted Metabolomic Analysis of Rhizosphere Soil

Non-targeted metabolomic profiling of rhizosphere soil samples from inoculated and control groups was performed using ultra-performance liquid chromatography-tandem mass spectrometry (UPLC-MS/MS). Freeze-dried soil samples were extracted with pre-cooled extraction solvent (methanol-acetonitrile-water, 2:2:1, *v*/*v*/*v*), vortexed, sonicated at low temperature, and centrifuged. The supernatants were filtered through membranes prior to analysis. Chromatographic separation was achieved on an ACQUITY UPLC BEH C18 column (2.1 mm × 100 mm, 1.7 μm) maintained at 40 °C, with gradient elution using 0.1% formic acid in water and 0.1% formic acid in acetonitrile as mobile phases. Mass spectrometric data were acquired in both positive and negative ion modes using an electrospray ionization (ESI) source.

Raw mass spectrometry data were converted using ProteoWizard (v3.0) software, and peak detection, alignment, and extraction were performed using the XCMS (v4.4.0) software package. Metabolites were identified by matching against databases including HMDB, MassBank, and METLIN. Following data preprocessing, statistical analyses were conducted: Student’s *t*-test (*p* < 0.05) combined with fold change criteria (|log_2_FC| > 1) was used to screen for differentially abundant metabolites between groups. Identified metabolites were annotated using the KEGG database, and their compositional characteristics were visualized using circular plots according to metabolite classes. Spearman’s rank correlation analysis was performed to explore relationships between relative abundances of differential metabolites and environmental factors. Additionally, scatter plots were generated to display the log_2_FC distribution of differential metabolites across compound classes, and bar plots were used to visualize the top 20 metabolites ranked by fold change.

## 3. Results

### 3.1. Selection of Microbial Consortia I and II

Based on comprehensive evaluation of plant growth-promoting traits, three bacterial strains—*Enterobacter* sp. 2, *Serratia* sp. 14, and *Bacillus* sp. 16—were selected to formulate bacterial consortium I for inoculation into *Lactuca sativa*. Among these, *Enterobacter* sp. 2 exhibited strong indole-3-acetic acid (IAA) production capacity, while *Serratia* sp. 14 demonstrated excellent phosphate solubilization, siderophore production, and IAA biosynthesis. Both *Enterobacter* sp. 2 and *Bacillus* sp. 16 showed ACC deaminase activity and moderate siderophore-producing capability (Supplementary Figure S1). For fungal consortium II, three fungal strains—*Penicillium* sp. 27, *Aspergillus* sp. 32, and *Talaromyces* sp. 33—were selected for inoculation into *S. nigrum*. Both *Penicillium* sp. 27 and *Talaromyces* sp. 33 exhibited pronounced inorganic and organic phosphate solubilization activities, whereas *Aspergillus* sp. 32 showed the lowest fermentation broth pH among all fungal strains evaluated (Supplementary Figure S1, Supplementary Table S1).

### 3.2. Effects of Microbial Consortia on Biomass and Yttrium Accumulation in L. sativa and S. nigrum

**Figure 1:**
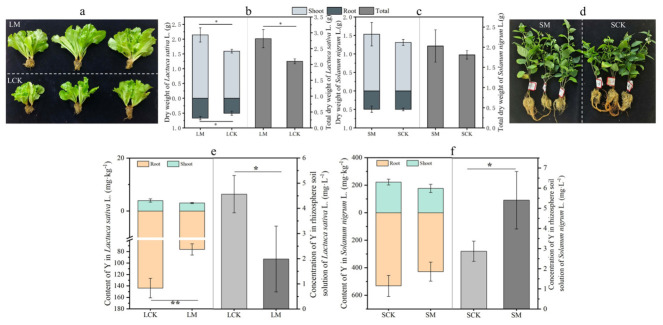
Effects of PGPR consortium I on *Lactuca sativa* and consortium II on *Solanum nigrum*: biomass and yttrium (Y) accumulation. (**a**) Growth phenotype of *L. sativa* after 30 days. (**b**) Dry weight of *L. sativa*. (**c**) Dry weight of *S. nigrum*. (**d**) Growth phenotype of *S. nigrum* after 30 days. (**e**) Y content in shoots, roots and rhizosphere soil solution of *L. sativa*. (**f**) Y content in shoots, roots and rhizosphere soil solution of *S. nigrum*. LCK: *L. sativa* non-inoculated control, LM: *L. sativa* inoculated with consortium I, SCK: *S. nigrum* non-inoculated control, SM: *S. nigrum* inoculated with consortium II. Data are presented as mean ± SD (*n* = 5). Asterisks indicate significant differences (* *p* < 0.05, ** *p* < 0.01) by LSD test.

Biomass measurements revealed that inoculation with bacterial consortium I significantly increased the shoot dry weight and total dry weight of *L. sativa* by 33% and 26%, respectively (Figure 1a,b). In contrast, no significant differences in either fresh or dry weight were observed between *S. nigrum* plants inoculated with fungal consortium II and their non-inoculated controls (Figure 1c,d).

Analysis of yttrium concentrations in plant roots and leaves, as well as in rhizosphere soil solutions, revealed markedly distinct regulatory patterns of the two microbial consortia on plant–yttrium interactions. Inoculation with consortium I significantly reduced yttrium content in *L. sativa* roots (by 47%, *p* < 0.01) and in the rhizosphere soil solution (by 56%, *p* < 0.05), while no significant effect was observed in aboveground tissues (Figure 1e). This finding suggests that consortium I may inhibit plant yttrium accumulation through modulation of yttrium bioavailability and regulation of uptake and transport systems. In striking contrast, inoculation with consortium II significantly increased yttrium concentration in the rhizosphere soil solution of *S. nigrum* by 89% (*p* < 0.05), yet this was not accompanied by a corresponding increase in plant tissue yttrium content (Figure 1f). This observation implies that while consortium II effectively mobilizes soil yttrium, the plants may simultaneously induce tolerance mechanisms to maintain yttrium homeostasis.

### 3.3. Effects of Microbial Consortia on Plant Stress Responses and Rhizosphere Soil Physicochemical Properties

**Figure 2:**
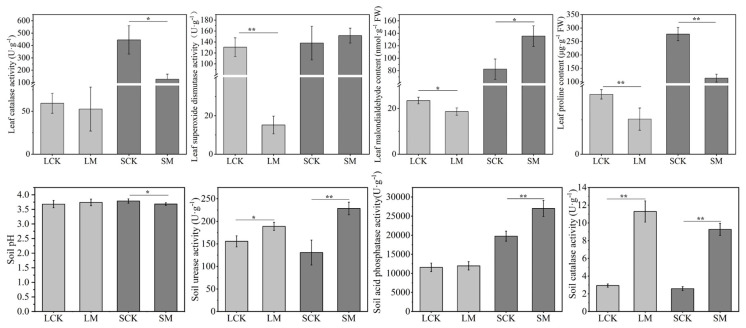
Effects of PGPR consortia on physiological and biochemical indicators in plant leaves and rhizosphere soil. (**Upper panels**) Proline (Pro) and malondialdehyde (MDA) contents (μg·g^−1^ FW), superoxide dismutase (SOD) and catalase (CAT) activities in leaves. (**Lower panels**) Soil pH, urease (S-UE), acid phosphatase (S-ACP) and catalase (S-CAT) activities (U·g^−1^) in rhizosphere soil. Data are presented as mean ± SD (n = 5). Asterisks indicate significant differences (* *p* < 0.05, ** *p* < 0.01) by LSD test.

Physiological indicator analysis revealed that both microbial consortia significantly alleviated stress in the host plants. Following inoculation with consortium I, *L. sativa* exhibited marked reductions in proline and MDA contents by 41% and 21%, respectively, accompanied by an 88% decrease in SOD activity (Figure 2). In *S. nigrum* inoculated with consortium II, proline content and CAT activity decreased by 59% and 72%, respectively (Figure 2). The substantial declines in osmoregulatory substances, oxidative damage markers, and antioxidant enzyme activities in both plant species indicate that the microbial consortia effectively mitigated rare earth element-induced stress.

Analysis of soil enzyme activities demonstrated that both consortia significantly enhanced rhizosphere microenvironment functionality. Inoculation with consortium I increased soil catalase and urease activities in the *L. sativa* rhizosphere by 285.32% and 21.40%, respectively (Figure 2). These enzymatic changes suggest that the bacterial consortium modulated key metabolic enzyme systems, thereby improving rhizosphere nutrient cycling and stress defense functions, ultimately creating a more favorable rhizosphere environment for plant growth. For *S. nigrum*, inoculation with consortium II reduced rhizosphere soil pH by 2.70%, a phenomenon potentially attributable to the acid-producing capability of *Aspergillus* sp. 32. Concurrently, significant enhancements were observed in soil catalase (259.69%), acid phosphatase (37.22%), and urease (74.62%) activities (Figure 2). Elevated catalase activity contributes to reactive oxygen species scavenging, thereby alleviating oxidative stress [20]; increased acid phosphatase facilitates organic phosphorus mineralization [21]; and enhanced urease activity promotes nitrogen cycling [22].

### 3.4. Effects of Microbial Consortia on Rhizosphere Soil Microbial Communities

**Figure 3:**
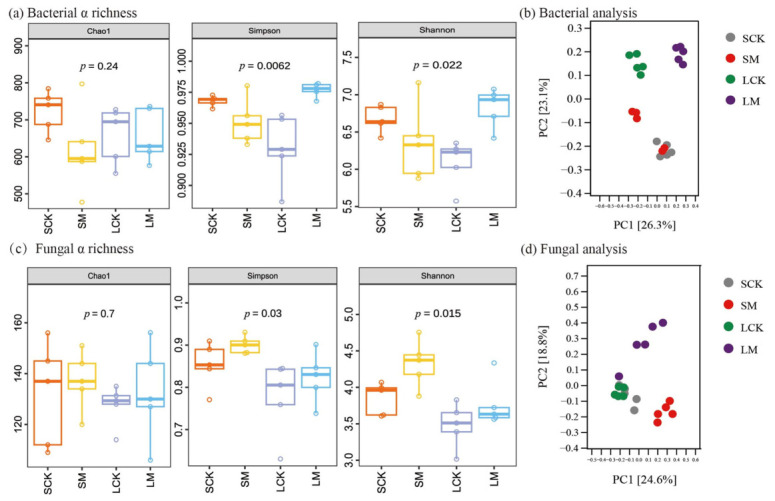
Alpha and beta diversity analyses of rhizosphere soil microbial communities. (**a**,**c**) Alpha diversity indices (Chao1, Simpson, and Shannon) for bacterial and fungal communities, respectively. circles represent individual biological replicates (*n* = 5). (**b**,**d**) Principal coordinate analysis (PCoA) plots based on Bray–Curtis distances depicting bacterial and fungal community structures, respectively. LCK: *L. sativa* non-inoculated control, LM: *L. sativa* inoculated with Consortium I, SCK: *S. nigrum* non-inoculated control, SM: *S. nigrum* inoculated with Consortium II.

Through comprehensive analysis of soil microbial community diversity, this study systematically evaluated the regulatory effects of consortia I and II on the rhizosphere microecology of *L. sativa* and *S. nigrum*, respectively. The results revealed distinct impacts of the different treatments on bacterial and fungal communities (Supplementary Figure S2). Alpha diversity analysis indicated that although no significant differences were observed in the Chao1 index for either bacterial or fungal communities across treatment groups (Figure 3a,c), microbial diversity exhibited consortium-specific regulatory patterns. Specifically, the bacterial diversity indices (Simpson and Shannon) were significantly higher in the *L. sativa* rhizosphere following inoculation with consortium I (LM) compared to the non-inoculated control (LCK). In contrast, fungal diversity indices were significantly elevated in the *S. nigrum* rhizosphere upon inoculation with consortium II (SM) relative to its control (SCK). These findings demonstrate that the microbial consortia effectively enhanced species evenness and ecological diversity within the rhizosphere microbial communities.

Principal coordinate analysis (PCoA) based on Bray–Curtis distances revealed clear separations between LCK and LM for bacterial communities, as well as between SCK and SM for fungal communities (Figure 3b,d). These distinct clusterings indicate that consortia I and II specifically modulated the bacterial community in the *L. sativa* rhizosphere and the fungal community in the *S. nigrum* rhizosphere, respectively.

### 3.5. Consortium I Modulates Rhizosphere Bacterial Community Structure and Enriches Functional Microbes to Promote L. sativa Growth

**Figure 4:**
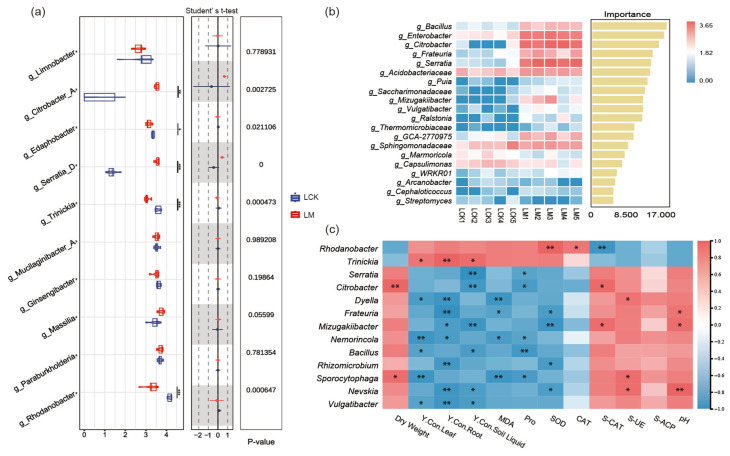
Key microbial taxa responsive to inoculation in *L. sativa* rhizosphere soil and their correlations with environmental factors. (**a**) Bacterial genera exhibiting significant abundance differences between LM (lettuce with consortium I) and LCK (lettuce control). (**b**) Importance ranking of microbial biomarkers identified by random forest analysis. (**c**) Correlation heatmap between differential microbial taxa (*y*-axis) and soil environmental factors (*x*-axis). Asterisks denote significant correlations (* *p* < 0.05, ** *p* < 0.01, *** *p* < 0.001).

Differential abundance analysis revealed that the relative abundances of *Citrobacter* and *Serratia* were significantly higher in the rhizosphere of inoculated *L. sativa* (LM) compared to the control (LCK) (Figure 4a). This finding indicates successful rhizosphere colonization by *Serratia* sp. 14 from the inoculated consortium.

Random forest analysis identified *Bacillus*, *Enterobacteriaceae*, *Citrobacter*, and *Serratia* as significant biomarkers in the rhizosphere soil of the inoculated group (LM) (Figure 4b). The emergence of these taxa as prominent biomarkers likely stems from the pivotal roles played by the three bacterial strains constituting consortium I, which collectively shaped the distinctive rhizosphere microbial community structure and thereby attained biomarker status.

Correlation analysis revealed that *Citrobacter* and *Sporocytophaga* exhibited significant positive correlations with plant dry weight and soil enzyme activities, while *Serratia* and *Citrobacter* showed positive associations with reduced yttrium concentrations in the rhizosphere soil solution (Figure 4c). These findings suggest that these genera may fulfill specific functional roles within the plant–soil system.

Notably, the microbial taxa showing significant positive correlations with plant dry weight were not the inoculated strains themselves, but rather *Citrobacter* and *Sporocytophaga*. *Citrobacter*, a recognized rhizosphere growth-promoting bacterium, may contribute through direct mechanisms including nitrogen fixation, phosphate solubilization, or phytohormone production [23,24]. *Sporocytophaga*, a cellulose-decomposing bacterium [25,26,27], suggests the involvement of indirect growth-promoting mechanisms: by degrading cellulose in the rhizosphere soil, this genus may improve soil structure and nutrient availability, thereby facilitating *L. sativa* growth. Collectively, these observations indicate that inoculation with consortium I may promote plant growth not solely through direct effects of the introduced strains, but also by modulating the rhizosphere microbial community to enrich indigenous populations with specific functional attributes.

### 3.6. Metabolite-Mediated Reduction of Yttrium Mobility and Plant Uptake by Consortium I in L. sativa

**Figure 5:**
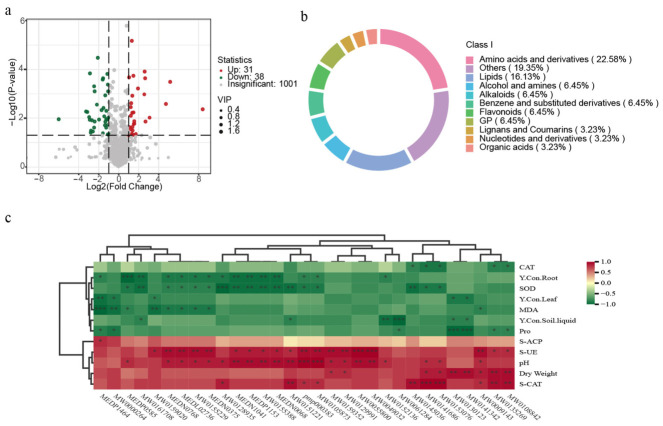
Rhizosphere metabolic profiling of *L. sativa* in response to consortium I inoculation. (**a**) Volcano plot of differentially abundant metabolites between LM (lettuce with consortium I) and LCK (lettuce control). (**b**) Circular plot showing compositional categories of upregulated metabolites. (**c**) Correlation heatmap between upregulated metabolites (*x*-axis) and environmental factors (*y*-axis). The color scale indicates Spearman correlation coefficients. Asterisks denote significant correlations (* *p* < 0.05, ** *p* < 0.01,*** *p* < 0.001).

Non-targeted metabolomic analysis revealed that inoculation with consortium I induced significant alterations in the rhizosphere metabolic profile of *L. sativa*. A total of 69 differentially abundant metabolites were identified in the rhizosphere soil of the inoculated group compared to the control, comprising 31 upregulated and 38 downregulated metabolites, while 1001 metabolites showed no significant changes (Figure 5a). These differential metabolites were primarily classified into amino acids and their derivatives, fatty acids, and other compound categories (Figure 5b).

Correlation analysis demonstrated that among the upregulated metabolites, laserpitin (MW0152136) and phosphatidylglycerol PG (a-13:0/i-12:0) (MW0061284) exhibited highly significant negative correlations with yttrium concentration in the rhizosphere soil solution. Additionally, deoxyuridine-phosphate (MW0161708), the dipeptide H-Phe-Phe-OH (MW0151221), dihydroquercetin (MW0130123), and croton factor F1 (MW0141342) showed significant negative correlations with rhizosphere solution yttrium levels (Figure 5c). These metabolites, which were substantially enriched in the inoculated group, may coordinately participate in the immobilization or transformation of yttrium ions within the rhizosphere solution, thereby contributing to the observed reduction in yttrium concentration.

Furthermore, yttrium accumulation in *L. sativa* roots exhibited a highly significant negative correlation with octadecatetraenoic acid (MEDP0585), and significant negative correlations with deoxyuridine-phosphate (MW0161708), octadecatrienoic acid (MEDN1041), tetracosatetraenoic acid (MEDP1153), the amino acid derivative Phe-Tyr-Lys-Arg (MW0155388), and homocysteine (MEDN0068) (Figure 5c). These significantly enriched rhizosphere metabolites—particularly unsaturated fatty acids (e.g., octadecatetraenoic acid, tetracosatetraenoic acid), sulfur-containing amino acids (e.g., homocysteine), and deoxyuridine-phosphate—may serve as effective organic ligands. Through their functional groups, including carboxyl, sulfhydryl, and phosphate moieties, these compounds can undergo complexation reactions with yttrium ions (Y^3+^), potentially forming stable metal–organic complexes [28,29,30,31]. This process may immobilize yttrium ions onto rhizosphere soil particles or root surfaces, or facilitate their transformation into less bioavailable forms (such as insoluble phosphate precipitates), thereby substantially reducing yttrium mobility and phytoavailability in the rhizosphere solution [32,33,34,35]. Consequently, this mechanism ultimately suppresses yttrium migration and accumulation from the rhizosphere into plant roots and aboveground tissues.

### 3.7. Rhizosphere Metabolic Reprogramming Induced by the Two Microbial Consortia

**Figure 6:**
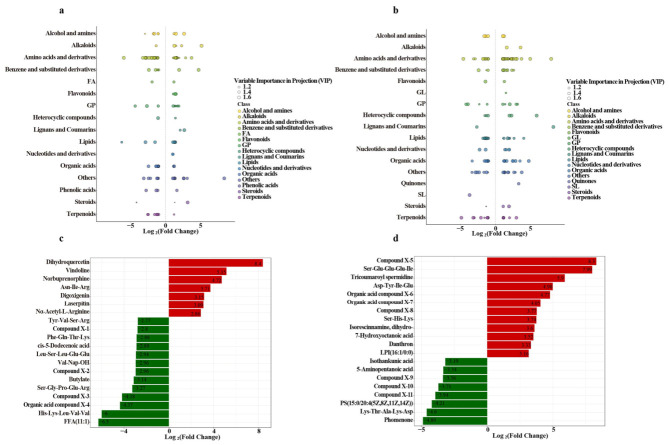
Distribution and fold change in differentially abundant metabolites. (**a**,**b**) Scatter plots showing the Log_2_FC distribution of differentially abundant metabolites across different compound classes in the *L. sativa* and *S. nigrum* rhizosphere soils, respectively. (**c**,**d**) Bar plots showing the top 20 metabolites ranked by fold change in the *L. sativa* and *S. nigrum* rhizosphere soils, respectively. The whole names of compounds X1–11 in the figure can be found in the Abbreviations section.

Non-targeted metabolomic analysis was performed on rhizosphere samples of *L. sativa* and *S. nigrum* following inoculation with their respective consortia (Figure 6a,b). In the *L. sativa* rhizosphere, consortium I upregulated flavonoids and alkaloids, and downregulated amino acids and their derivatives, organic acids, and fatty acids (Figure 6a,c). In the *S. nigrum* rhizosphere, consortium II upregulated amino acids and their derivatives, organic acids, lignans, and coumarins (Figure 6b,d). As shown in Table 1, the majority of differentially expressed amino acids (e.g., Ser-His-Lys, Trp-Ala-Phe) and organic acids (e.g., sulfonated derivatives, 3-hydroxydecanoic acid) in the *S. nigrum* rhizosphere were upregulated following consortium II inoculation.

## 4. Discussion

### 4.1. Microbial Consortia Alleviate Plant Stress and Influence Growth Through Divergent Pathways

Consortia I and II effectively mitigated rare earth element-induced stress in *L. sativa* and *S. nigrum*, respectively, yet their effects on plant growth exhibited contrasting patterns. In *L. sativa*, inoculation with consortium I significantly reduced leaf proline and malondialdehyde contents (by 41% and 21%, respectively), indicating effective alleviation of physiological stress, concomitantly with a 26% increase in biomass. This growth promotion likely arose from two synergistic factors: direct plant growth-promoting activities of the inoculated strains (e.g., IAA production, ACC deaminase activity, phosphate solubilization, siderophore production), and the reshaping of rhizosphere bacterial communities by consortium I, which enriched indigenous functional taxa such as *Citrobacter* and *Sporocytophaga*. These taxa, identified as key biomarkers by random forest analysis and exhibiting significant positive correlations with plant dry weight, may promote growth indirectly through mechanisms including cellulose decomposition, soil structure improvement, or nutrient mobilization [36,37]. Collectively, these findings elucidate a multifaceted growth-promoting pathway whereby inoculation with consortium I not only provides direct beneficial effects through the introduced strains but also modulates the indigenous microbial community to enrich functional taxa that further contribute to plant growth.

In contrast, although fungal consortium II similarly alleviated oxidative stress in *S. nigrum* (evidenced by a 59% reduction in proline content), it did not significantly enhance biomass. This discrepancy likely stems from differences in consortium composition and the inherent biological characteristics of the constituent strains. Consortium I comprises three bacterial strains with well-characterized plant growth-promoting traits (e.g., IAA production, ACC deaminase activity, phosphate solubilization, siderophore production), rendering its mechanisms inherently predisposed toward direct growth promotion and stress alleviation. Conversely, the fungal strains in consortium II exhibited few typical plant growth-promoting features. With the exception of phosphate solubilization capacities displayed by *Penicillium* sp. 27 and *Talaromyces* sp. 33, these strains lacked other common plant growth-promoting traits. Their most pronounced characteristic was instead the strongly acidic nature of their fermentation broths. Consequently, the primary influence of consortium II on plants may be mediated through metabolic activities (such as acid production) that substantially alter the rhizosphere chemical environment, rather than through direct stimulation of plant growth. This fundamental distinction in rhizosphere interaction modes aligns with the divergent design objectives of the two consortia: consortium I was intended to promote growth while immobilizing yttrium in the rhizosphere, whereas consortium II was designed to mobilize the rhizosphere metal pool.

### 4.2. Directional Remodeling of Rhizosphere Microbial Communities and Metabolic Networks

The present study revealed that the two microbial consortia differentially modulated the rhizosphere microflora of their respective host plants, providing the ecological basis for their functional divergence. Bacterial consortium I primarily altered the bacterial community structure in the *L. sativa* rhizosphere (as evidenced by significant separation in PCoA analysis) and enriched biomarker genera including *Enterobacter* and *Serratia*. In contrast, fungal consortium II significantly enhanced fungal diversity (as indicated by increased Shannon index) and reshaped the fungal community structure in the *S. nigrum* rhizosphere. This inoculant-type-specific regulation, whereby bacterial consortium I predominantly influenced bacterial communities and fungal consortium II targeted fungal communities, may be attributable to the inherent characteristics of the inoculants themselves, as well as the distinct microecological niche preferences associated with different plant rhizospheres.

Furthermore, these shifts in community structure were directly linked to alterations in functional metabolic networks [38,39]. Correlation analysis revealed that in the *L. sativa* rhizosphere, genera such as *Citrobacter* and *Serratia* exhibited significant negative correlations with yttrium concentrations in the soil solution. This suggests that the enrichment or activation of these functionally specialized populations following consortium I inoculation may drive localized biogeochemical processes, ultimately leading to yttrium immobilization. Conversely, consortium II substantially enhanced fungal communities in the *S. nigrum* rhizosphere. This modulation, combined with the acid-producing capacity and phosphate solubilization activities of the constituent fungi, may collectively contribute to a microbial functional network conducive to metal mobilization. This coupling between community structure and function ultimately reshapes the rhizosphere chemical environment (including pH and metabolic exudates), thereby influencing yttrium bioavailability in the rhizosphere.

### 4.3. Core Metabolite-Mediated Mechanisms of Yttrium Bioavailability in the Rhizosphere

The distinct rhizosphere metabolic reprogramming induced by the two consortia, as detailed in Figure 6, provides the chemical basis for the differential regulation of yttrium behavior. In the *L. sativa* rhizosphere, consortium I induced an immobilization-oriented metabolic pattern characterized by global upregulation of flavonoids and alkaloids, accompanied by widespread downregulation of amino acids and their derivatives, organic acids, and fatty acids. Dihydroquercetin—the metabolite exhibiting the highest fold change in the *L. sativa* rhizosphere—is a flavonol with potent antioxidant properties that can significantly enhance plant tolerance to oxidative stress [40]. More importantly, its ortho-phenolic hydroxyl groups possess strong metal-chelating capacity, potentially immobilizing yttrium ions (Y^3+^) through the formation of stable metal–organic complexes, thereby reducing their bioavailability [41,42,43]. Vindoline, the metabolite with the second highest fold change, is a plant defense-related alkaloid. Its enrichment suggests activation of systemic resistance responses in *L. sativa* [44,45]. Concurrently, amino acid derivatives and organic acids—which can form soluble complexes with metals—were predominantly downregulated, reducing the supply of metal-mobilizing agents. This dual metabolic remodeling, characterized by upregulation of immobilizing factors and downregulation of mobilizing factors, provides a chemical basis for the substantial reduction in yttrium concentration observed in the *L. sativa* rhizosphere solution and the consequent decrease in root uptake.

In the *S. nigrum* rhizosphere, consortium II induced a mobilization-oriented metabolic pattern dominated by substantial upregulation of amino acids and their derivatives, organic acids, lignans, and coumarins. Among these, Compound X-5—a coumarin derivative—was the most highly upregulated metabolite and has been associated with plant defense against metal stress. Certain coumarins are known to influence metal transport and detoxification through complexation mechanisms [46,47]. Organic acids, including various sulfonated derivatives and 3-hydroxydecanoic acid, function as canonical metal-mobilizing agents: their carboxyl groups form soluble complexes with Y^3+^, thereby enhancing metal mobility in the soil solution [48,49]. Table 1 further confirms the widespread upregulation of amino acids and organic acids in the *S. nigrum* rhizosphere after consortium II inoculation. This widespread enrichment of organic acids and amino acid derivatives may represent the primary driver of yttrium mobilization in the rhizosphere. The coordinated accumulation of these metabolites not only likely contributed to the observed decrease in rhizosphere soil pH but also enhanced Y^3+^ solubility and chemical reactivity through the provision of abundant organic ligands. These findings mechanistically explain the significant increase in rhizosphere yttrium concentration and establish favorable rhizosphere chemical conditions for potential phytoextraction.

Collectively, these metabolomic profiles suggest a potential chemical basis for the differential yttrium behavior observed in the two rhizosphere systems. In the *L. sativa* rhizosphere, the upregulation of metal-chelating flavonoids (e.g., dihydroquercetin) together with the downregulation of metal-mobilizing organic acids may contribute to yttrium immobilization. In the *S. nigrum* rhizosphere, the upregulation of organic acids and coumarin derivatives may promote yttrium complexation and solubility, potentially enhancing its bioavailability in the soil solution. However, given the complexity of rhizosphere processes, further investigation is needed to elucidate the direct causal links between specific metabolites and yttrium speciation.

## 5. Conclusions

This study demonstrates that specific microbial consortia can precisely regulate the rhizosphere behavior of yttrium.

Bacterial consortium I reshaped the bacterial community structure in the *L. sativa* rhizosphere. It also altered the metabolic profile, upregulating flavonoids and alkaloids while downregulating organic acids. These changes promoted yttrium immobilization and reduced plant uptake. The consortium simultaneously alleviated stress and enhanced plant growth.

Fungal consortium II enriched acid-producing fungi in the *S. nigrum* rhizosphere. This drove substantial upregulation of organic acids and amino acids. These metabolic shifts effectively mobilized the rhizosphere yttrium pool, establishing a foundation for potential resource recovery.

Together, these findings provide a theoretical and technical basis for developing microbe-mediated differential remediation strategies for rare earth element-contaminated sites.

## Figures and Tables

**Table 1:** Differential metabolites (amino acids and derivatives, organic acids) in *S. nigrum* rhizosphere soil following consortium II inoculation.

Compounds	SM_vs_SCK_*p*-Value	SM_vs_SCK_Fold_ Change	SM_vs_SCK_Type
Amino acid and derivatives			
Ser-His-Lys	2.40 × 10^−2^	13.34	up
Trp-Ala-Phe	2.76 × 10^−2^	8.34	up
Glu-Phe-Leu-Val-Met	1.27 × 10^−2^	7.28	up
N2-(1-Carboxyethyl)-L-arginine	1.59 × 10^−2^	5.58	up
Arg-Pro-Ser	3.22 × 10^−2^	5.57	up
Asn-Ile-Arg	1.78 × 10^−3^	5.52	up
Lys-Phe-Phe	3.09 × 10^−2^	3.50	up
Lys-His-Ala	3.09 × 10^−3^	3.36	up
Asn-Ile-Phe-Lys	1.15 × 10^−2^	2.75	up
Lys-Thr-Ile-Thr-Leu	3.96 × 10^−2^	2.41	up
His-Lys-Leu-Val-Val	1.64 × 10^−4^	2.40	up
Phe-TyrMe-OH	1.84 × 10^−3^	2.15	up
Phe-HoPhe-OH	1.62 × 10^−2^	2.09	up
Gln-Glu-Asp	2.20 × 10^−4^	2.09	up
Glutathione Reducedform	7.72 × 10^−3^	0.47	down
His-Phe-His	2.57 × 10^−4^	0.37	down
Ile-Phe-Arg-Lys	3.75 × 10^−2^	0.31	down
Ile-Val	5.10 × 10^−6^	0.29	down
Glutamylproline	2.84 × 10^−2^	0.13	down
Glu-Val	8.44 × 10^−3^	0.11	down
Lys-Thr-Ala-Lys-Asp	1.13 × 10^−4^	0.04	down
Organic acids			
6-{3,5-dihydroxy-2-[(2E)-1-hydroxy-3-(4-hydroxyphenyl)prop-2-en-1-yl]-4-{[3,4,5-trihydroxy-6-(hydroxymethyl)oxan-2-yl]oxy}phenoxy}-3,4,5-trihydroxyoxane-2-carboxylic acid	1.59 × 10^−2^	27.37	up
6-{3,5-dihydroxy-4-[3,4,5-trihydroxy-6-(hydroxymethyl)oxan-2-yl]phenoxy}-3,4,5-trihydroxyoxane-2-carboxylic acid	3.63 × 10^−3^	7.32	up
[2-(2-hydroxypropan-2-yl)-6-(2-methylbut-3-en-2-yl)-7-oxo-2H,3H,7H-furo[3,2-g]chromen-3-yl]oxidanesulfonic acid	2.59 × 10^−3^	5.55	up
[2,6-dihydroxy-4-(3,5,7-trihydroxy-3,4-dihydro-2H-1-benzopyran-2-yl)phenyl]oxidanesulfonic acid	2.16 × 10^−3^	3.04	up
3-[3,4-dihydroxy-5-(3,4,5-trihydroxybenzoyloxy)benzoyloxy]-5-hydroxy-4-methoxybenzoic acid	2.19 × 10^−3^	2.48	up
{4-[(1E)-3-(4-methoxyphenyl)-3-oxoprop-1-en-1-yl]phenyl}oxidanesulfonic acid	2.69 × 10^−3^	2.40	up
3-Hydroxydecanoic acid	1.17 × 10^−2^	2.30	up
{8-[2-(acetyloxy)propan-2-yl]-2-oxo-2H,8H,9H-furo[2,3-h]chromen-9-yl}oxidanesulfonic acid	7.00 × 10^−5^	0.46	down
{4-[3,5-dihydroxy-8-(hydroxymethyl)-8-methyl-4-oxo-4H,8H-pyrano[2,3-f]chromen-2-yl]phenyl}oxidanesulfonic acid	3.13 × 10^−5^	0.38	down

## Data Availability

The original contributions presented in this study are included in the article/Supplementary Material. Further inquiries can be directed to the corresponding author.

## Supplementary Materials

The following supporting information can be downloaded at: https://www.mdpi.com/article/10.3390/microorganisms14050962/s1, Figure S1: Functional characterization of plant growth-promoting traits of the tested strains. (a–c) Phosphorus solubilization: (a) *Serratia* sp. 14, (b) *Penicillium* sp. 27, (c) *Talaromyces* sp. 33 (top: inorganic P; bottom: organic P). (d,e) Siderophore production: (d) *Enterobacter* sp. 2, (e) *Serratia* sp. 14. (f,g) IAA production: (f) *Enterobacter* sp. 2 (top left) and *Bacillus* sp. 16 (top right); (g) *Serratia* sp. 14 (top right); controls shown at bottom. (h,i) ACC deaminase production: (h) *Enterobacter* sp. 2, (i) *Bacillus* sp. 16.; Figure S2: Soil microbial community structure in *Lactuca sativa* and *Solanum nigrum* rhizosphere; Table S1: pH of fermentation broths from plant growth-promoting bacteria (LB) and fungi (PDB).

## Abbreviations

The following are abbreviations for some compounds in Figure 6:

Compound X-1	2,3-(S)-hexahydroxydiphenoyl-D-glucose
Compound X-2	Sphingosine-1-phosphocholine
Compound X-3	(25S)-5ß-spirostan-3ß-yl-ß-D-glucopyranosyl-(1->3)-[ß-D-xylopyranosyl-(1->4)-ß-D-glucopyranosyl-(1->4)]-ß-D-glucopyranoside
Compound X-4	(2S)-2-amino-3-[hydroxy-[(2R)-2-[(Z)-octadec-11-enoyl]oxy-3-pentadecanoyloxypropo-xy]phosphoryl]oxypropanoic acid
Organic acid Compound X-5	8-[2-(acetyloxy)-1-hydroxypropan-2-yl]-2-oxo-2H,8H,9H-furo[2,3-h]chro-men-9-yl(2E)-2-methylbut-2-enoate
Organic acid Compound X-6	6-{3,5-dihydroxy-2-[(2E)-1-hydroxy-3-(4-hydroxyphenyl)prop-2-en-1-yl]-4-{[3,4,5-trihydroxy-6-(hydroxymethyl)oxan-2-yl]oxy}phenoxy}-3,4,5-trihydroxyoxane-2-carboxylic acid
Organic acid Compound X-7	15S-hydroperoxy-11Z,13E-eicosadienoic acid
Compound X-8	alpha-Pyrrolidinopropiophenone
Compound X-9	(3S,8S,9R,10R,13R,14S,17R)-17-[(2R)-5,6-dihydroxy-6-methylheptan-2-yl]-3-hydroxy-44,9,13,14-pentamethyl-1,2,3,7,8,10,12,15,16,17-decahydrocyclopenta[a]phenanthren-11-one
Compound X-10	N-(dodecanoyl)-sphing-4-enine-1-phosphocholine
Compound X-11	[[(2R,3S,4R,5R)-5-(4-amino-2-oxopyrimidin-1-yl)-3,4-dihydroxyoxolan-2-yl]methoxy-hydroxyphosphoryl][(2S)-2,3-bis(3,7,11,15-tetramethylhexadecoxy)propyl] hydrogenphosphate
