# Supplementary material for: Microbial-Mediated Differential Regulation of Yttrium Behavior in the Rhizosphere: Blocking Uptake in Lactuca sativa L. While Enhancing Bioavailability in Solanum nigrum L
Source: Microorganisms. 2026 Apr 24;14(5):962. doi: 10.3390/microorganisms14050962 (PMC13209652; doi:10.3390/microorganisms14050962)
Supplement: Supplementary file 1 [file microorganisms-14-00962-s001.zip › Supplemental Table S1.pdf]

Supplemental Table S1 pH of fermentation broths from plant growth-promoting bacteria (LB) and fungi (PDB).

Microorganisms	pH
Enterobacter sp. 2	8.9
Serratia sp. 14	8.2
Bacillus sp. 16	9.26
Penicillium sp. 27	4.11
Aspergillus sp. 32	1.95
Talaromyces sp. 33	3.9
