# Supplementary figures and images for: Microbial-Mediated Differential Regulation of Yttrium Behavior in the Rhizosphere: Blocking Uptake in Lactuca sativa L. While Enhancing Bioavailability in Solanum nigrum L
Source: Microorganisms. 2026 Apr 24;14(5):962. doi: 10.3390/microorganisms14050962 (PMC13209652; doi:10.3390/microorganisms14050962)

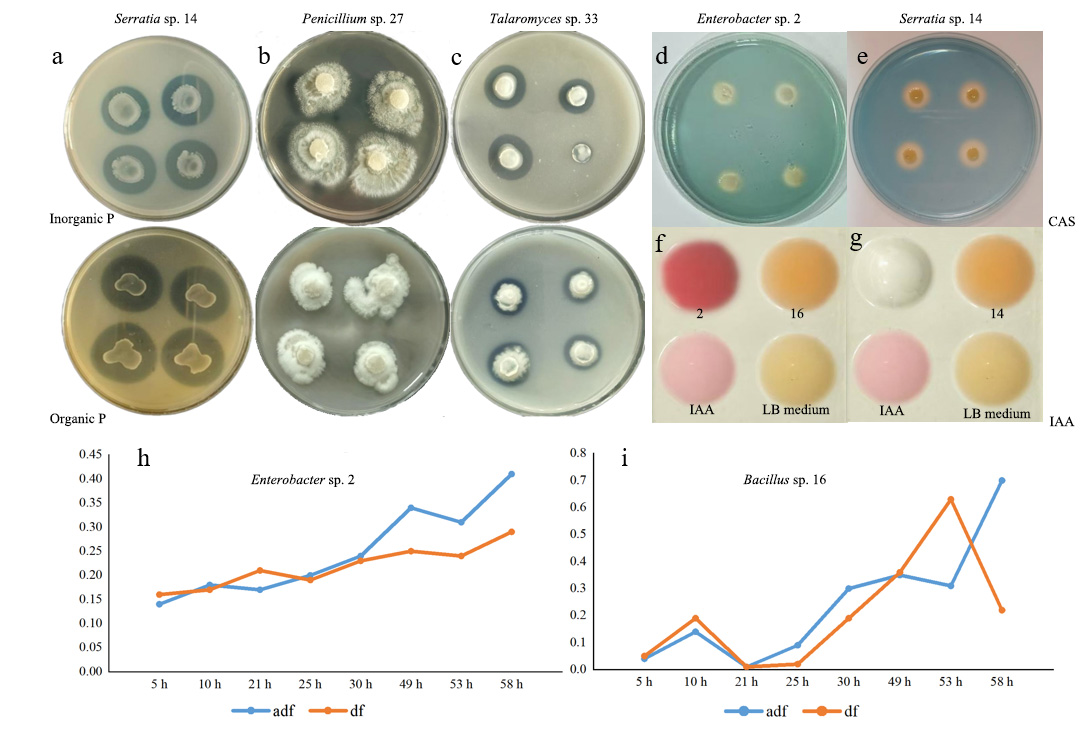

Supplement: Supplementary file 1 [file microorganisms-14-00962-s001.zip › Supplemental Figure S1.jpg]

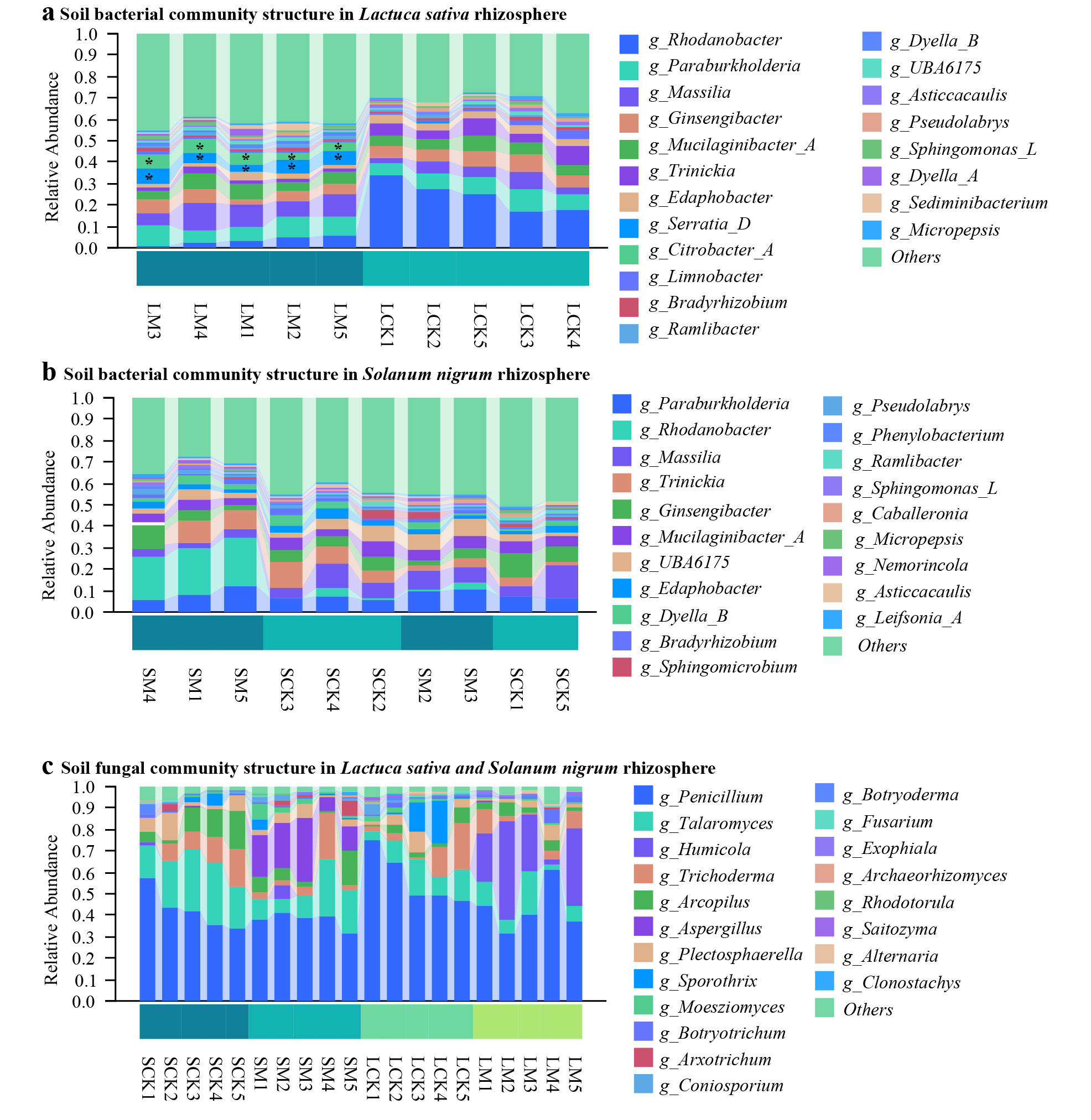

Supplement: Supplementary file 1 [file microorganisms-14-00962-s001.zip › Supplemental Figure S2.jpg]
